# Effect of Hemp Shive Granulometry on the Thermal Conductivity of Hemp–Lime Composites

**DOI:** 10.3390/ma18153458

**Published:** 2025-07-23

**Authors:** Wojciech Piątkiewicz, Piotr Narloch, Zuzanna Wólczyńska, Joanna Mańczak

**Affiliations:** Faculty of Civil Engineering, Warsaw University of Technology, Al. Armii Ludowej 16, 00-637 Warsaw, Poland

**Keywords:** hemp–lime composite, hemp shives, granulometry, thermal conductivity, particle size distribution, binder-to-shive ratio, image analysis, sustainable building material

## Abstract

This study investigates the effect of hemp shive granulometry on the thermal conductivity and microstructure of hemp–lime composites. Three distinct particle size fractions—fine, medium, and coarse—were characterized using high-resolution image analysis to determine geometric parameters such as Feret diameters, circularity, and elongation. Composite mixtures with varying binder-to-shive and water-to-shive ratios were prepared and compacted at a consistent level to isolate the influence of aggregate granulometry on thermal performance. Results demonstrate a clear inverse relationship between particle size and thermal conductivity, with coarse fractions reducing thermal conductivity by up to 7.6% compared to fine fractions. Composite density was also affected, decreasing with increasing particle size, confirming the impact of granulometry on pore structure and packing density. However, binder content exhibited the most significant effect on thermal conductivity, with a 20% increase observed for higher binder-to-shive ratios irrespective of shive size. The study further establishes that a 15 g sample size (~2400 particles) provides sufficient statistical accuracy for granulometric characterization using image analysis. These findings provide critical insights for optimizing hemp–lime composites for enhanced thermal insulation performance, supporting sustainable construction practices by informing material formulation and processing parameters.

## 1. Introduction

### 1.1. Effect of Hemp Shive Size on Thermal Conductivity

Hemp–lime composite is a lightweight construction material obtained by combining an organic aggregate—hemp shives (the woody inner parts of the hemp stalk)—with a lime-based binder. It is characterized by very high porosity, which provides excellent thermal insulation properties [1,2]. Typical thermal conductivity values (λ) for dry hemp–lime composites range from approximately 0.06 to 0.13 W/(m·K), depending on bulk density, mix composition, and measurement orientation [3,4]. Due to its hygroscopic and open-pore structure, the hemp–lime composite also helps regulate indoor humidity and microclimate, offering an attractive and ecological alternative to conventional insulation materials [5].

**Figure 1:**
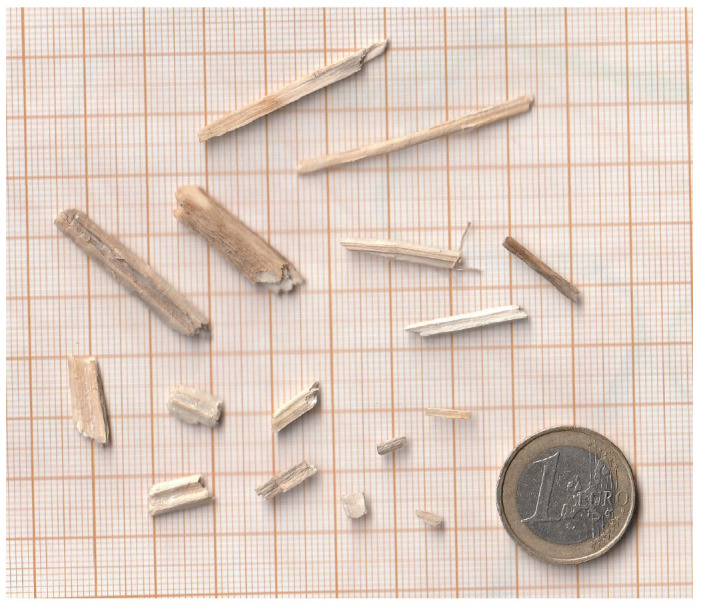
Irregularity of the shape of hemp shives used in the study.

The size of hemp shives can be adjusted through cultivation methods (e.g., sowing density) and processing technologies (e.g., roller tooth geometry used for decortication). Due to the anatomical structure of hemp, longitudinal particles aligned with the plant’s growth direction are formed during crushing and decortication [5]. Their length is typically several times greater than their width (see Figure 1). An increased proportion of fine shive fractions leads to a denser composite structure. Smaller particles more effectively fill the voids between larger ones, which increases the bulk density and reduces the overall porosity of the material compared to mixtures using coarser aggregate. Experimental research confirms this correlation: Fine fractions increase the composite’s density and decrease its porosity. For example, in starch-based hemp composites, the use of short shives (0–5 mm) resulted in significantly higher bulk density and lower porosity compared to composites with coarse shives (0–20 mm) [6]. The bulk density of the fine-fraction sample was approximately 135 kg/m^3^, with a porosity of ~89%, whereas the coarse-fraction composite had a density of ~110 kg/m^3^ and a porosity of ~91% [6]. Similarly, another study found that reducing the average shive size (from ~9 mm to ~3 mm) led to a noticeable decrease in the total porosity of the resulting hemp composite [7]. Smaller particles fill more free space in the mix, reducing the volume of air voids. Consequently, composites with higher content of fine fractions exhibit greater packing density and lower porosity, which often translates to changes in other properties (e.g., mechanical or thermal) [7].

Finer hemp shive fractions have a greater specific surface area compared to coarser fractions. This means that for the same filler volume, smaller particles require more binder coverage and absorb more mixing water. Shives smaller than 5 mm increase the demand for both water and binder due to their higher surface area [4].

The same pattern holds for binders other than lime: Larger hemp shives produce more porous mixtures, reduce the material’s density, and promote better thermal insulation. Finer shives can fill part of the voids, slightly increasing composite density and thermal conductivity (λ), even when binder content remains constant [8].

In one study [9], a composite made with fine hemp shives (FHS) exhibited a thermal conductivity of λ = 0.1050 W/(m·K), while that made with coarse shives (THS) achieved λ = 0.0992 W/(m·K)—a difference of around 6%, attributed to the slightly higher bulk density of the fine-shive composite (approximately 382 kg/m^3^ vs. 377 kg/m^3^). Williams et al. [4] studied thermal conductivity of composites using shives with mean lengths of ~7.5 mm (medium fraction) and ~15.3 mm (coarse fraction). A slightly higher λ was observed for the finer fraction, with both values around 0.12 W/(m·K); the differences were minimal. The authors noted that the effect of granulometry was less significant than expected—fine shives increased λ only marginally.

In another study [4], French hemp shives were divided into four fractions: 7 (fine), 14 (coarse), and 2 blended fractions (8 and 12, mixed 50/50 by weight, medium). Particle size was determined via image analysis of 20 g of aggregate in ImageJ, measuring bulk density, median particle length and width, interquartile range, and mean aspect ratio (length/width). The analysis assumed equal particle density and that width equaled thickness, ignoring irregular shapes and varying bulk densities. Results showed minimal influence of shive size on thermal conductivity. For mixtures with a binder-to-shive ratio of 2.2, thermal conductivity measured perpendicular to the casting direction was about 0.121 W/(m·K) for fine aggregate and about 0.118 W/(m·K) for coarse, suggesting only a slight improvement in insulating performance with increased particle size. However, when the casting direction was parallel to heat flow, λ dropped to about 0.1 W/(m·K) for the fine fraction—indicating a 17% reduction. A similar trend was observed for coarse shives, highlighting that composite anisotropy has a more pronounced impact on thermal insulation than particle size. Other studies also confirm that shive granulometry has a minor influence on λ—typically only a few percent—compared to other factors [4,9,10,11].

Some researchers aim to identify the optimal balance between short and long shive particles. In study [6], composites were tested using either 0–5 mm (fine) or 0–20 mm (coarse) shives. The fine fraction had a higher bulk density (~135 kg/m^3^) and slightly lower porosity (89.3% vs. 91.3%) than the coarse fraction. In single-fraction samples, the composite made entirely of fine shives was denser and exhibited slightly higher thermal conductivity than that made of coarse shives. To achieve an optimal balance of mechanical and hygrothermal properties, a mix containing 30% fine (0–5 mm) and 70% coarse (0–20 mm) shives was developed. This blend achieved the best thermal properties while maintaining minimum strength requirements [6].

**Figure 2:**
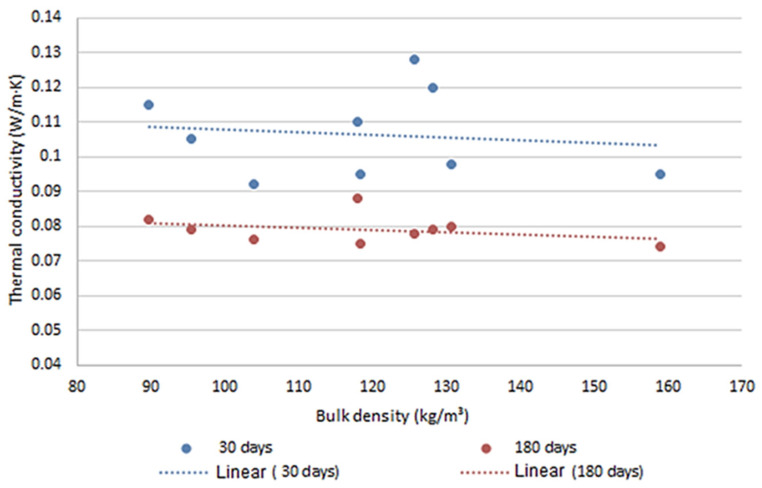
Influence of hemp shives and sample age on thermal conductivity.

There are, however, studies reporting significantly greater impact of shive granulometry on thermal conductivity. A comprehensive study [7] tested 13 different types of hemp shives with bulk densities ranging from 70.8 kg/m^3^ to 158 kg/m^3^ (Figure 2). When processed using the same production method (compression, casting direction), the same binder, and the same curing conditions, thermal insulation performance varied noticeably. Samples tested at 30 days exhibited differences of up to 34% (λ = 0.092 to 0.123 W/(m·K)). At 180 days, the difference decreased but remained significant—up to 27%.

In starch- and shive-based insulation boards, the smallest shive fraction (2.5–5 mm) enabled the lowest thermal conductivity, λ ≈ 0.061 W/(m·K) for dry material with a density of approximately 280 kg/m^3^. In comparison, samples containing larger particles (fractions of 5–20 mm) exhibited higher λ values in the range of 0.07–0.09 W/(m·K) at similar densities. The finest shives allowed for slightly better insulation performance than mixtures dominated by coarser fractions [12].

Hemp shives primarily consist of three biopolymers: cellulose, hemicellulose, and lignin. Additionally, pectins—mainly present in the plant—along with lipids, carbohydrates, and proteins, play a significant role when using plant aggregates in combination with mineral binders. These compounds interact with cement and lime components, weakening the binding process [5]. The chemical composition of hemp can vary depending on climatic conditions, harvest timing, processing methods, and plant variety. In study [13], hemp shives produced in France, Germany, and the United Kingdom were compared. The plant aggregates from these countries differed in cultivar, harvest date and method, and decortication technique (Table 1). Therefore, assessing the influence of hemp shive granulometry based on materials from different suppliers may be misleading and fail to account for chemical composition differences.

### 1.2. Influence of Component Proportions on Thermal Conductivity

Hemp shives, which constitute the primary component of hemp–lime composites, exhibit relatively low thermal conductivity due to their porous nature, typically ranging from 0.051 to 0.058 W/m·K [14]. In contrast, the mineral binder forming the composite matrix possesses significantly poorer thermal insulation properties. The principal component of the binder—hydrated lime—has a thermal conductivity in the range of 0.65 to 0.84 W/m·K in its dry mortar form [15]. Therefore, determining the binder-to-shive mass ratio is a critical factor in the design of hemp–lime composites. Reported values in the literature vary widely, from as high as 4.82 [10] to as low as 0.69 [16]. For cast-in-place wall applications (shuttering method), commonly used ratios fall within the range of 1.5 to 2 [17,18]. In a comparative study [19] evaluating four different binder-to-shive ratios from 0.75 to 2.5, a significant increase in both composite density and thermal conductivity—up to 66%—was observed.

**Figure 3:**
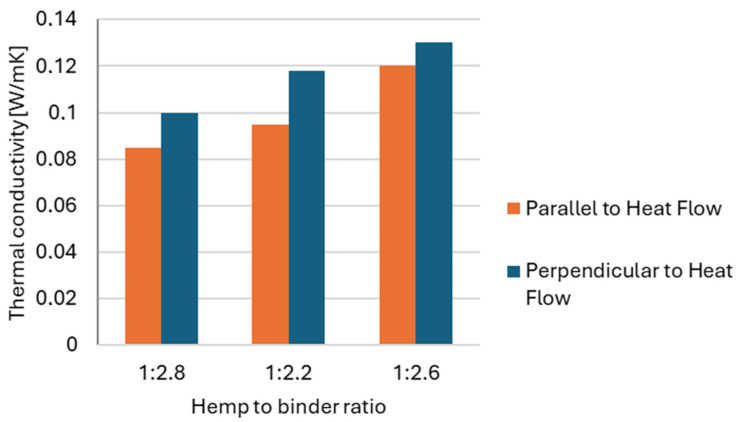
Thermal conductivity of samples with three shive-to-binder ratios, formed in two orientations [4].

Another parameter that significantly affects material density is the water-to-shive ratio. The optimal amount of water is rarely defined precisely in the literature; instead, practitioners often rely on empirical assessments of mix workability or practical experience [20,21]. Nevertheless, it is well established that water content influences the compaction behavior and the resulting density of the mixture. As noted by [22], wet mixes are more readily compacted because the water-absorbed aggregate becomes heavier, more elastic, and more deformable. Study [4] also demonstrated that the binder content in the mix exerts a considerably stronger effect on the composite’s thermal performance than aggregate granulometry. An increase in the binder-to-shive ratio from 1.8 to 2.6 resulted in a 29% rise in thermal conductivity for composites containing medium-sized shives (see Figure 3).

### 1.3. Influence of Material Compression on Thermal Conductivity

In the literature, various descriptions can be found regarding the method and degree of compaction, such as “light tamping” [4], “manual compaction using a wooden mallet” [9], or “ramming to eliminate large air voids” [23]. Studies [24,25] investigated the influence of compaction on the mechanical and thermal properties of hemp–lime composites. In these studies, fresh material was compressed under stress ranging from 0.6 to 1 MPa, reducing its initial volume by a factor of 3. By varying the level of compaction, researchers observed a significant improvement in mechanical properties, accompanied by a moderate reduction in thermal conductivity due to decreased material porosity [24].

In another study [26], three levels of compaction were defined based on the percentage reduction in volume relative to the uncompressed state: 30%, 45%, and 60%. The compaction level directly influenced both compressive strength and thermal conductivity. A similar approach was adopted in study [4], where samples were cast in 50 mm layers, and compaction was quantified as a 45% densification relative to the loose-state density. The loose-state density was determined by weighing a known volume of uncompressed material gently placed by hand.

It is important to note that the 45% value in [26] refers to volume reduction, whereas in [4], it refers to increased density. Thus, the same percentage value does not denote the same physical process. According to the terminology in [26], a 45% volume reduction corresponds to a final volume of approximately 68.97% of the initial volume.

### 1.4. Methods for Determining Hemp Shive Size

The most commonly reported characteristics of hemp shives used in hemp–lime composites are bulk density and origin (see Table 2). Bulk density provides a general indication of particle size and shape, while the material’s origin may reflect the processing technology, and thus, the quality of the aggregate (e.g., fiber and dust content and granulometric uniformity) [27]. Nevertheless, details regarding the presence of fibers and fines—which can significantly affect composite properties—are often omitted or limited to vague statements.

Another important property that is sometimes reported or investigated is the water absorption capacity of hemp shives. This parameter influences the amount of water required in the mix to ensure proper binder hydration, as well as the drying time of the material and the potential risk of biological degradation [28].

Among the most frequently provided parameters by hemp shive suppliers is the particle size distribution. According to French hemp construction association standards, this distribution defines the size range within which 95% of the particles fall [29]. However, this range is often very broad and does not provide a precise representation of the actual particle dimensions. Furthermore, hemp shives exhibit an elongated shape, meaning that reporting only a single dimension can be misleading. Occasionally, granulometry is described using a generalized dimension (e.g., 5 × 5 × 15 mm) [30], a broad range of geometric attributes, or a vague reference to particle length [31].

**Table 2:** Characteristics of hemp shives in scientific literature.

Origin	Bulk Density	Absolute Density	Porosity	Water Absorption	Mean Particle Length	Mean Particle Width	Mean Particle Thickness	Length Range	Width Range	Thickness Range	Major Length	Major Width	Width to Length Ratio		Source
X	X				X	X									[26]
X	X				X	X	X								[10]
X	X							X	X	X					[31]
	X														[30]
X	X														[32]
X	X	X	X	X									X		[33]
X	X														[34]
X	X			X											[35]
X	X			X											[36]
X	X			X											[16]
X	X														[37]
X	X			X											[38]
X	X														[39]
X					X	X					X	X			[9]
X	X														[27]
X	X				X	X									[40]
X	X				X	X							X		[4]
X	X			X	X	X									[7]
X	X			X											[41]
	X	X	X	X											[25]
**Width to Length Ratio Graph**	**Particle Size Graph**	**Equivalent Diameter**	**Minor and Major Axis**	**Elongation**	**Particle Size Range**	**Grading Curve**	**Fiber Content**	**Dust Content**	**Particle Dimensions**	**Particle Surface area**	**Mean Particle Surface Area**	**Mean Particle Mass**	**Fraction**	**Extractive Proportions**	**Reference**
															[26]
															[10]
					X		X	X							[31]
									X						[30]
					X		X	X							[32]
X															[33]
					X										[34]
	X	X	X												[35]
							X	X							[36]
		X	X	X					X	X					[16]
															[37]
		X	X							X					[38]
						X	X								[39]
													X		[9]
						X	X	X							[27]
						X									[40]
						X									[4]
				X							X	X			[7]
		X	X					X		X				X	[41]
						X									[25]

**Figure 4:**
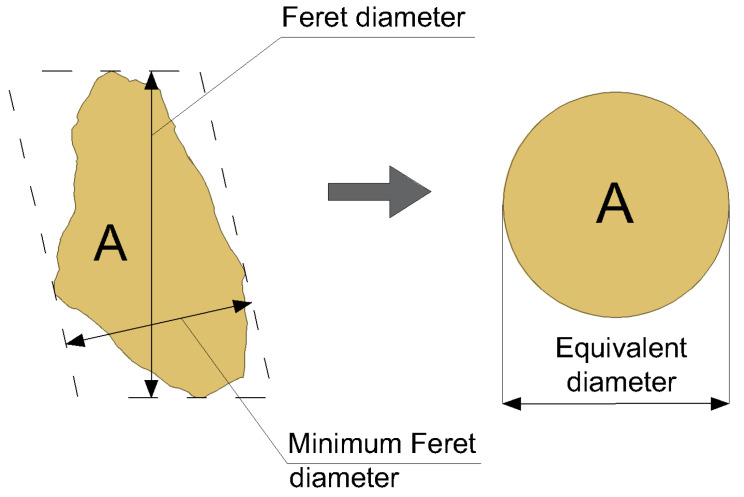
Definition of geometric parameters used to characterize the shape of hemp shives.

To precisely describe the shape of hemp shives, researchers most commonly report a set of dimensional parameters: the average particle length and width, the major and minor axes of an ellipse fitted to the aggregate particle, or the maximum and minimum Feret diameters (see Figure 4).

There is no clearly defined standard for determining the length and width of irregularly shaped particles such as hemp shives. These dimensions are most commonly obtained through image analysis techniques [16,35,36,41]. Many researchers manually measure a batch of several grams of particles using tools such as calipers, which is a highly time-consuming task due to the small size of the aggregate [39]. Based on these measurements, parameters such as particle elongation—defined as the ratio of the major to minor axis [7,16]—or the width-to-length ratio [4,33] can be calculated.

Image analysis also enables accurate measurement of the surface area of each individual particle. From these data, researchers often derive the equivalent diameter, defined as the diameter of a circle having the same area as the measured particle [16] (see Figure 4).

Another useful parameter for describing particle shape is circularity, where a value of 0 corresponds to an infinitely elongated shape and a value of 1 represents a perfect sphere [16]. Circularity is calculated using the following Equation (1):

(1) $$Circularity=\frac{4\pi \cdot A}{P^{2}}$$

where *A* is the particle surface area and *P* is the perimeter.

Reporting only the average size of hemp shives is often insufficient due to the high variability in particle surface areas. Therefore, researchers frequently use graphical methods to illustrate the distribution of particle characteristics, such as elongation, circularity, or size (e.g., length, width, or axes) [4]. Rather than referencing particle count, these analyses typically rely on the cumulative area represented by the particles [41]. This approach provides a clearer representation of how specific sizes or shapes are distributed across the total volume (or surface area) of the aggregate.

In recent years, increased focus on sustainable construction has driven research into composite materials derived from renewable resources. The RILEM Technical Committee TC 236-BBM (2010–2016) developed recommendations for characterizing bio-aggregates, with a particular emphasis on hemp shives. The committee highlighted that traditional sieve analysis is less suitable for these materials due to their irregular, elongated, and porous particle structure, which can lead to inaccurate granulometric classification. Instead, it recommends advanced imaging and morphological analysis, such as digital image-based techniques, for precise determination of particle dimensions and shapes. While sieve analysis may be used as a comparative method with clearly defined testing conditions, the committee advocates adopting modern measurement techniques to enhance result repeatability and reliability [42].

The various available methods for measuring the geometric characteristics of hemp shives differ significantly in terms of data resolution, accuracy, and experimental requirements (see Table 3). Sieve analysis enables the examination of relatively large sample masses—for example, 100 g of hemp shives were sieved in [34], and up to 112 g in [39]. Such quantities contain tens of thousands of particles, providing a statistically stable and representative granulometric distribution. However, the main source of uncertainty in this method is that irregular particles can pass through sieve openings based on their orientation, not actual dimensions. Elongated fragments may be classified into smaller fractions if they pass vertically through the mesh despite their longer dimension. Consequently, sieve analysis primarily reflects particle width rather than true length or shape [32,34,39]. Furthermore, intensive sieving may cause fragile particles to break apart, increasing the proportion of fine fractions (dust).

A non-invasive method of size determination is manual measurement using calipers. This method is inherently time-consuming and typically includes hundreds of individual particles. For example, in study [9], over 1000 fragments per sample were manually measured for length and width. Such high numbers are rare; more often, researchers limit the sample to a few hundred or fewer representatively selected particles, which may compromise the accuracy of the size distribution. Additionally, manual caliper measurements are subject to human error. Consistently applying the caliper to each irregular particle is challenging, and selecting what constitutes “length” or “width” may be subjective (e.g., whether to include protruding fibers). Although digital calipers typically have high resolution (0.01 mm), human error and fatigue over time can result in deviations of several tenths of a millimeter.

Two-dimensional (2D) image analysis methods—based on digital processing of scanned or photographed particles—allow for the automated determination of length, width, projected area, and shape parameters for each individual particle. These methods offer a more representative depiction of particle geometry than traditional sieving. Studies have confirmed that granulometric curves obtained via sieve analysis and 2D image analysis differ substantially [25]. Relying solely on sieve methods for plant-based aggregates can result in imprecise observations, whereas image analysis provides a more accurate description of particle morphology [38].

Software such as ImageJ enables automatic measurement of hundreds or thousands of particles from relatively small samples (typically 3–20 g) [4,33,38]. Due to the low density of hemp shives, even small sample masses translate into large particle counts. According to RILEM TC 236-BBM guidelines, image analysis should use 3–6 g of shives, which corresponds to at least 2000 particles—a quantity deemed sufficient to achieve a statistically representative size distribution [42]. Increasing the number of particles enhances statistical reliability but also increases the workload of data preparation and processing.

Proper image preparation and processing are crucial for 2D image analysis. Overlapping or touching particles may be interpreted as a single large particle, inflating the measured dimensions. The RILEM protocol recommends placing hemp shives flat and separated on the scanner surface. Another major source of error is the thresholding (binarization) level used to segment particles. If the threshold is too low, thin particle edges may be lost; if it is too high, adjacent particles may merge or appear oversized due to halo effects. Careful selection of the threshold level is therefore critical [33]. Dust and very fine particles may introduce noise, distorting the distribution by contributing a large number of small objects.

One limitation of 2D image analysis is that it does not capture particle thickness (the third dimension). Estimating volume or 3D shape requires additional assumptions or independent thickness measurements. For example, in [4], particle volume was approximated by assuming that thickness is proportional to particle width. While this assumption allows rough volume estimation, it introduces uncertainty and simplifies the real particle geometry. More accurate 3D characterization of hemp shives can be obtained via X-ray computed tomography (CT) [10]. This method enables 3D reconstruction of particle morphology, providing precise measurements of size, volume, porosity, and shape factors. Despite its accuracy, CT is limited by high equipment costs, the need for specialized personnel, long scan times at high resolutions, sample preparation constraints, and the computational demands of 3D data analysis. As such, it remains impractical for routine use, especially in studies with large sample sizes or limited resources.

Therefore, the aim of this study is to assess the influence of hemp shive granulometry on the thermal conductivity of hemp–lime composites under controlled conditions, using image-based aggregate characterization.

## 2. Materials and Methods

### 2.1. Materials

#### 2.1.1. Binder Characteristics

The binder was prepared using hydrated lime (CL 90-S), Portland cement (CEM I 42.5R), and metakaolin. The proportions and composition of the binder were selected to utilize materials widely available in Poland and to allow for a precise characterization of its chemical makeup. Based on previous studies, a composition consisting of 70–75% hydrated lime, 15–20% hydraulic binder, and 10–15% pozzolanic additive is among the most commonly used formulations [3,10,19,31,43,44,45,46,47].

The addition of cement is intended to improve early strength, which is important for practical applications such as rapid formwork removal. The inclusion of a pozzolanic material such as metakaolin serves to reduce thermal conductivity while also having a lower embodied energy compared to cement [33,48,49]. By reacting with calcium hydroxide, metakaolin forms cementitious compounds, which enhance the durability and mechanical strength of the binder system [50].

The specific proportions of additives in the lime-based binder were chosen to optimize binder reactivity and the mechanical performance of the hemp–lime composite material [34].

#### 2.1.2. Hemp Shives

Industrial hemp of the Futura 75 variety, commonly used for construction purposes [27,51,52], was used in this study. The crop was grown during a single vegetation season (2023) and processed by the same manufacturer—Podlaskie Konopie (Dobrzyniówka, Poland)—into fiber and hemp shives. This ensured the uniformity of the raw material and minimized the influence of variables other than particle size distribution. During the production process, the hemp underwent natural retting, a method in which cut hemp stalks are left in the field, where moisture and weather conditions (fog, rain, temperature) promote colonization by microorganisms, primarily fungi. These microorganisms penetrate the stalk and break down the pectins that bind the fiber to the woody core [53]. This process, controlled by the farmer, serves as a form of preliminary decortication, which facilitates the separation of fibers from the woody part used to produce shives. A side effect of retting is the localized discoloration of some hemp shive particles.

The delivered hemp shives were separated into fractions using mechanical sieving with 2 mm and 4 mm mesh screens. This resulted in two size fractions: fine (F), consisting of particles passing through the 4 mm sieve and retained on the 2 mm sieve, and coarse (C), comprising particles retained on the 4 mm sieve. A third fraction, medium (M), was prepared as a 1:1 weight mixture of the fine and coarse fractions. The bulk density of the prepared aggregate groups was determined in accordance with the recommendations of RILEM TC 236-BBM (Table 4) [42].

**Figure 5:**
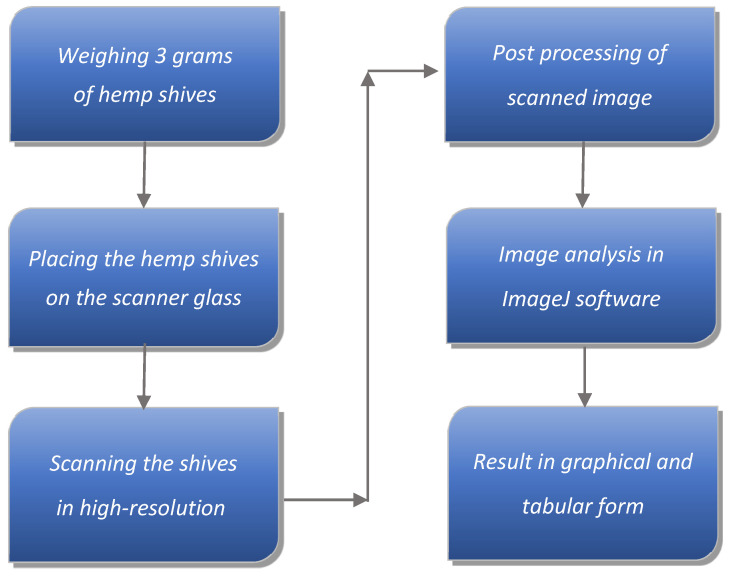
Schematic diagram of hemp shive particle size determination.

An image analysis method was employed to precisely determine particle shapes and accurately characterize each granulometric fraction. The applied algorithm (see Figure 5), designed for detailed analysis of hemp shive particle sizes in composite mixtures, involved conducting 30 independent scans of 3 g samples. Before each scan, the material was manually spread on a flatbed scanner within an area of approximately 180 × 180 mm (±3 mm), ensuring that individual particles did not touch each other. This arrangement was essential to enable unambiguous contour detection and to minimize image analysis errors.

Samples were scanned at a 1200 × 1200 dpi resolution in 24-bit color mode, and the images were saved in lossless PNG format. This configuration ensured high image quality, resolution, and contrast, which are critical for precise segmentation and subsequent data processing in ImageJ software, version 1.54g (National Institutes of Health, Bethesda, MD, USA). Further in the manuscript, the software is referred to simply as *ImageJ*. To reduce segmentation errors caused by particle color variability, a supervised image correction step was implemented, allowing for high-accuracy contour identification.

The final images were imported into the ImageJ environment, calibrated using a reference scale, and analyzed using the “Analyze Particles” function. This process yielded fundamental geometric parameters of the particles, such as area, perimeter, Feret diameter, and minimum Feret diameter. The Feret diameter represents the maximum dimension of a particle and can be interpreted as its maximum length, given the lack of a strict definition for particle length. Conversely, the minimum Feret diameter can be considered equivalent to the maximum particle width.

**Figure 6:**
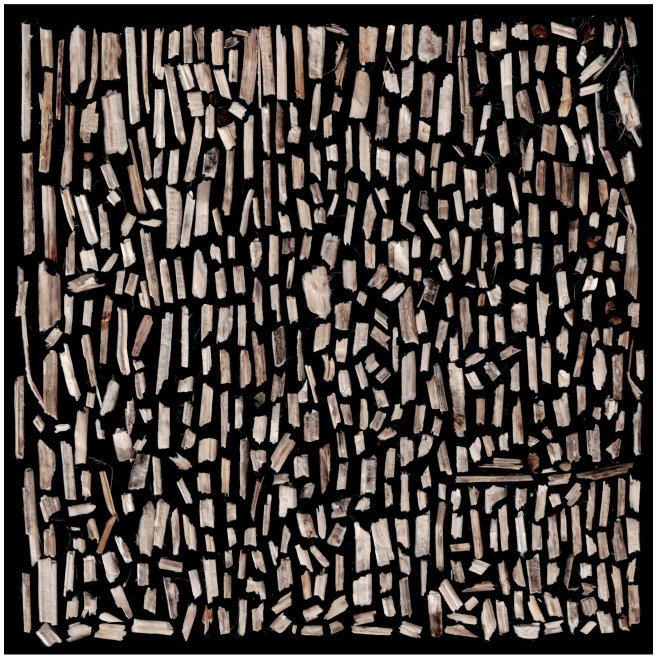
Sample scan of 3 g of hemp shives arranged on a 180 × 180 mm surface.

**Figure 7:**
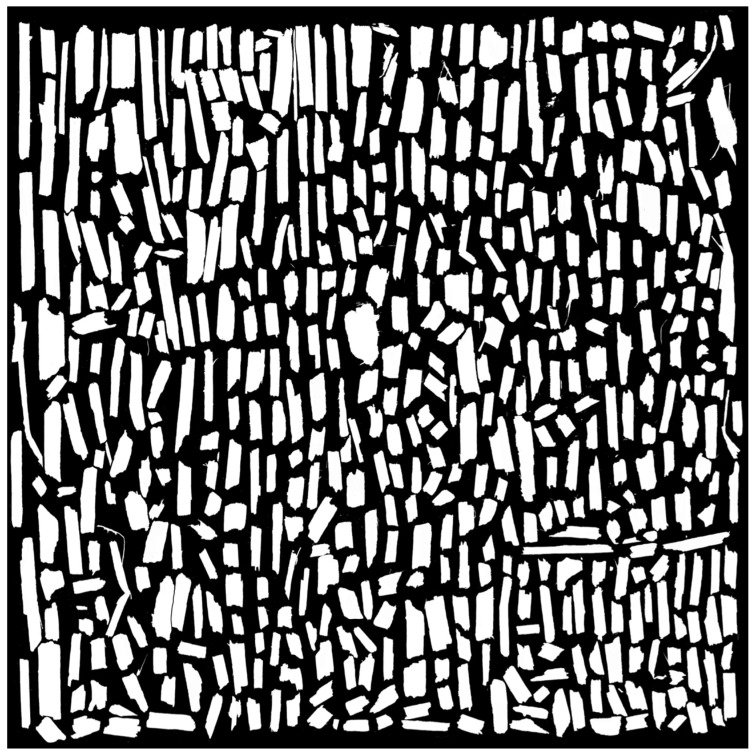
Left: thresholded binary images showing the material structure. Right: detected contours of the analyzed structures in ImageJ.

**Figure 8:**
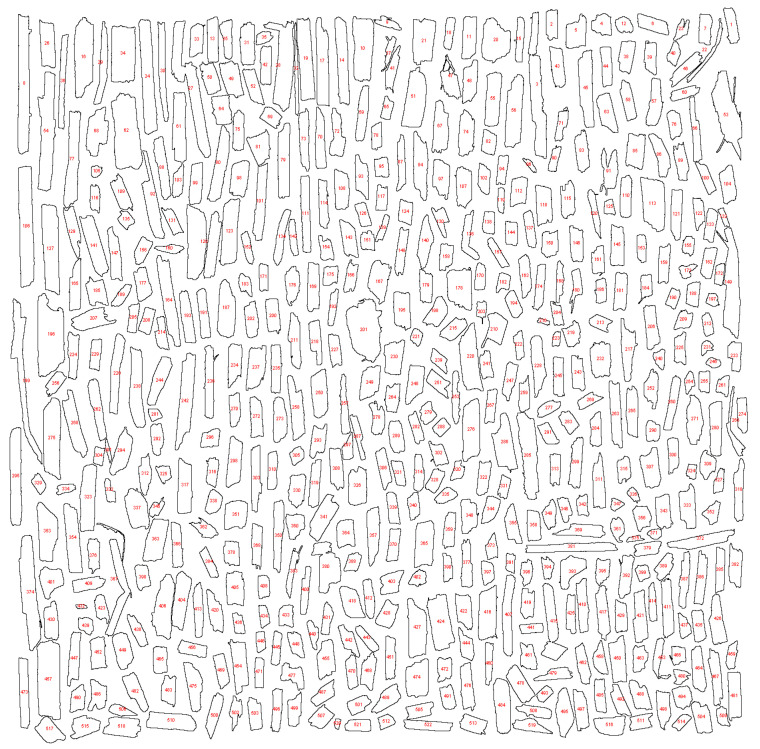
Identification of hemp shive particles using ImageJ (red numbers denote particle IDs).

To ensure transparency and enable reproducibility, all high-resolution scanned images have been made publicly available on Zenodo (DOI: 10.5281/zenodo.15664512, accessed on 22 July 2025) [54]. Due to their large size, these files were not included directly as Supplementary Materials. The Zenodo repository also contains the binary images obtained through thresholding, as well as detailed morphometric analysis results exported from ImageJ in spreadsheet format (*.xlsx), including a comprehensive summary of all scans. Below, selected illustrative examples are presented: a scan of a 3 g hemp shive sample (Figure 6), the corresponding binary image (Figure 7), and the contours of the analyzed particles with individual numbering (Figure 8).

**Figure 9:**
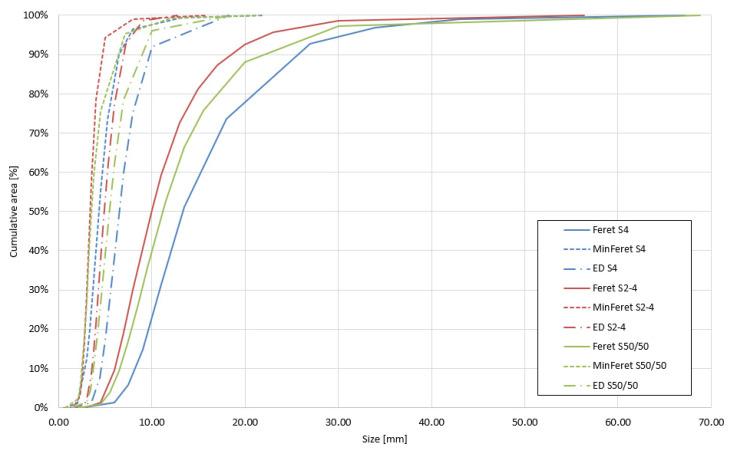
Particle size distribution of hemp shives.

Image analysis provided a comprehensive dataset enabling precise characterization of particle shape and size. Table 4 presents a set of geometric parameters calculated based on the aggregated ImageJ results for each granulometric group. To accurately illustrate the particle size distribution, graphs were generated showing the maximum Feret diameter, minimum Feret diameter, and equivalent diameter (ED) in relation to the percentage contribution of each class to the total particle surface area (see Figure 9).

### 2.2. Experimental Design

To assess the influence of hemp shive granulometry on the thermal conductivity of hemp–lime composites, a series of laboratory mixtures was developed, varying in terms of aggregate particle size distribution and the proportions of hemp shives, water, and binder (see Table 5). Each mixture was prepared and tested under controlled conditions to enable a detailed analysis of the relationship between material structure and thermal performance.

The formulation was based on one of the most commonly adopted shive-to-binder mass ratios—2:1—frequently cited in the literature on hemp–lime composites [3,9,19,21,30,31,35,36,43,44,46,47,49,52,55,56,57,58].

Given the lack of a universally accepted method for determining the optimal water content in hemp–lime mixtures, the adopted values were derived from a review of previous studies employing similar binder compositions. In studies [31,43,52,55], for mixtures with comparable binder content, the mass ratio of water to shives ranged from 2.75 to 2.9. In a doctoral dissertation [20], where a binder consisting of 70% hydrated lime (CL90-S), 15% Portland cement (CEM II/B-V 32.5 R), and 15% metakaolin was used, the binder–shive–water ratio was set at 2:1:2.8.

Based on literature data as well as preliminary experimental observations, a water-to-shive mass ratio of 2.74 was selected for this study. In the second tested mixture, a higher binder content was introduced to evaluate its impact on the thermal conductivity of the composite. In all cases, the same compaction level of the fresh mix was applied—150% relative to its loose-state volume—to ensure uniform sample preparation conditions.

### 2.3. Methods

#### Sample Preparation

In the first stage of sample preparation, appropriate amounts of all composite components—binder constituents, hemp shives, and water—were accurately weighed. The dry binder ingredients were then thoroughly mixed, after which the entire volume of water was added and blended using a planetary mixer with a vertical rotation axis. This preparation method—starting with the premixing of the binder and water—has also been employed in other experimental studies on hemp–lime composites [10,18,59].

After achieving a homogeneous binder consistency (typically within 1–3 min), the hemp shives were gradually added to the mixer. Due to the low bulk density of the shives, this specific sequence of component addition was necessary to prevent overloading the mixer and to avoid the risk of material dispersal beyond the mixing chamber. Once the binder was evenly distributed throughout the shive mass, the mixing process was considered complete, and the mixer was turned off. The total mixing time ranged from 7 to 9 min.

Immediately after preparation, the bulk (loose) density of the mixture—i.e., without compaction—was determined using a box-shaped mold measuring 30 × 30 cm with a height of 8 cm.

After determining the loose bulk density of the mixture in the laboratory and setting the target compaction level to 150%, the required material mass for a single sample was calculated. Samples were formed in molds of the same dimensions as those used for bulk density assessment, but oriented vertically (30 cm in height), using a wooden tamper. The mixture was compacted in four equal layers, each approximately 7.5 cm thick, ensuring uniform densification. The total mixing time did not exceed 5 min. A comparable procedure was applied in [4], where the compaction degree was defined as a 45% densification of the loose-state density, equivalent to 145% according to the present study’s methodology.

**Figure 10:**
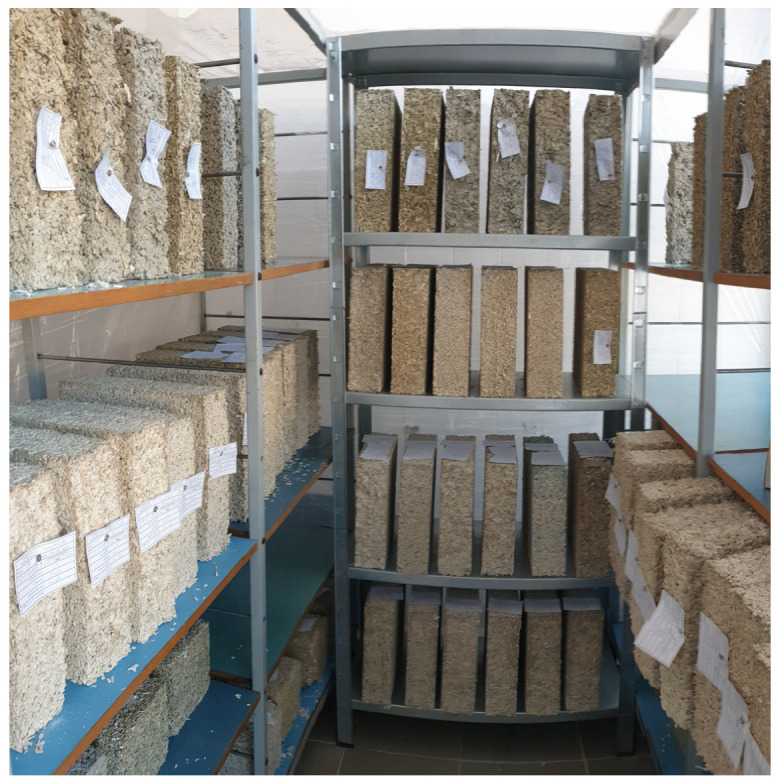
Samples from each series prepared for thermal conductivity testing.

After 24 h, the samples were demolded and transferred to a climate chamber maintained at 20 ± 2 °C and 60 ± 10% relative humidity (RH). All material property tests were conducted after a 120-day curing period. Prior to testing, the specimens were further conditioned for 7 days under laboratory conditions at 20 ± 2 °C and 50 ± 10% RH (see Figure 10). The samples were tested in an air-dry state, with a stabilized moisture content of approximately 12% (by weight), measured after curing and conditioning.

**Figure 11:**
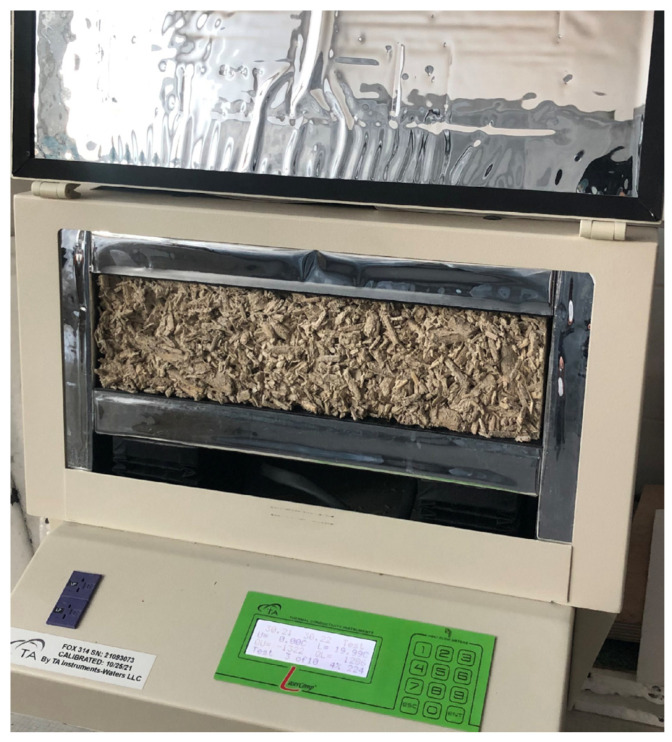
Thermal conductivity testing using the FOX 314 apparatus.

Thermal conductivity was measured using a FOX 314 heat flow meter (manufacturer: TA Instruments, New Castle, DE, USA) with a measurement area of 10 × 10 cm (Figure 11), based on the principle of unidirectional, homogeneous, and steady-state heat transfer. The operation of the device relies on the one-dimensional Fourier’s law, as expressed in Equation (2):

(2) $$q=-\lambda \frac{dT}{dx}$$

where:

*q*—heat flux (W/m^2^),
*λ*—thermal conductivity (W/m·K),
*dT*/*dx*—temperature gradient (K/m).

The samples were placed between two flat plates with controlled temperatures—a heated lower plate and a cooled upper plate—with a temperature difference of 20 K and an average sample temperature of 20 °C, in accordance with EN 12,667 and the procedure described in [19]. Heat flow occurred vertically—from bottom to top—in a direction perpendicular to the compaction direction of the sample, reflecting the actual heat transfer conditions in shuttered wall assemblies, which replicate the construction method used on-site (see Figure 11). Each measurement lasted at least 3 h, and five independent samples were tested for each material series.

## 3. Results

### 3.1. Sample Density

**Figure 12:**
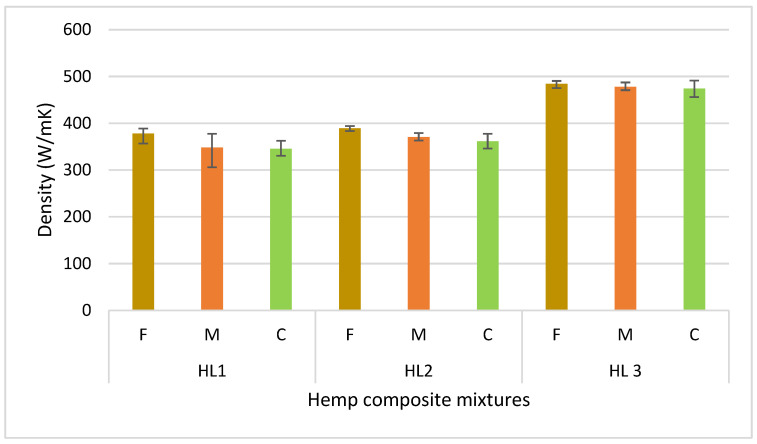
Average density of samples from each mixture.

In all series, as the aggregate size increased, a noticeable decrease in sample density was observed (Figure 12). The density drop between fine (F) and coarse (C) aggregates ranged from 2.0% to 8.6%. The most significant decrease occurred in the HL1 series (with lower binder content), while the smallest was observed in HL3 (with higher binder content). The density difference between fine (F) and medium (M) aggregates was more pronounced than between medium (M) and coarse (C) ones. In the HL1 series, the density decreased by as much as 7.9%, while in HL2, the reduction was 4.9%. Further density decreases between medium and coarse aggregates were less significant, ranging from 0.8% to 2.4%.

An increase in the water content of the mixture resulted in higher composite density. The most noticeable density increase was observed in the series with medium aggregate, where the difference between HL1.M and HL2.M was 6.4%. The smallest increase occurred in the mixtures with fine aggregate, with a difference of only 3% between HL1.F and HL2.F. On average, increasing the water-to-binder ratio from 1.37 to 1.57 resulted in a density increase of 4.7%.

### 3.2. Thermal Conductivity

**Figure 13:**
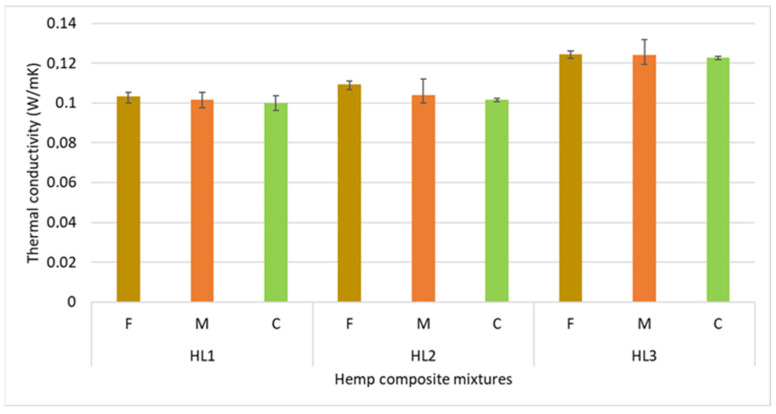
Thermal conductivity (λ) of hemp–lime composite mixtures as a function of shive granulometry and binder-to-shive ratio.

Similar to the trends observed in sample density, an increase in the average particle size of hemp shives resulted in a decrease in thermal conductivity (Figure 13). The most pronounced drop was observed in series HL2, where thermal conductivity decreased by 7.6% between the fine and coarse aggregates. Comparable decreases were recorded in series HL1 and HL3, amounting to 3.3% and 1.5%, respectively.

The difference in λ between fine (F), medium (M), and coarse (C) fractions is not consistent across all series. For instance, the drop in thermal conductivity between HL2F and HL2M was 5.2%, whereas between HL3F and HL3M, it was only 0.3%. The most proportional reduction in λ was observed in series HL1, where the stepwise differences between consecutive particle sizes were 1.7% and 1.6%, respectively. The lowest thermal conductivity was achieved in series HL1C at 0.1000 W/m·K, while the highest was observed in HL3F at 0.1246 W/m·K.

An increase in water content in the mixture improved compressibility and resulted in a higher thermal conductivity after curing. This effect was most visible for the fine aggregate, with a 5.7% increase. For medium and coarse aggregates, the changes were more modest—2.2% and 1.5%, respectively.

The most significant factor influencing thermal conductivity was the binder-to-shive ratio. For each particle size, increasing the binder content resulted in an increase in λ of over 20%. The highest increase was observed in the coarse aggregate series (22.8%) followed closely by the medium aggregate series (22.2%). For fine aggregate mixtures, thermal conductivity increased from 0.1033 W/m·K at a 2:1 binder-to-shive ratio to 0.1246 W/m·K at a 3:1 ratio—an increase of 20.5%.

## 4. Discussion

In study [55], which employed nearly identical binder composition, mixing ratios, and hemp shives from the same supplier as the present work, the composite density reached 497.9 kg/m^3^. In contrast, in the present study, the density of samples varied between 346 and 378 kg/m^3^, depending on the shive granulometry. Although both studies used a tamping method that simulates the shuttering technique applied on construction sites, the resulting densities differed significantly, indicating substantial variation in the degree of compaction during sample formation. Despite the more than 30% higher density achieved in [55], the reported thermal conductivity was only marginally higher—0.108 W/m·K—measured in the dry state.

In contrast, studies [10,31,52], which used binders of similar composition (hydrated lime, hydraulic binder, and pozzolanic additives) and nearly identical shive–binder–water ratios as those used in series HL1, reported composite densities in the range of 388–394 kg/m^3^. This can be attributed to slightly higher compaction compared to the 150% compaction level adopted in the present work. Notably, none of these studies explicitly stated the degree of compaction, generally limiting the description to a recommendation for careful tamping to minimize air voids and ensure material uniformity. At such densities, thermal conductivity values ranging from 0.098 to 0.106 W/m·K were reported [31,52], which aligns well with the values obtained for HL1 and HL2. This comparison suggests that the hemp shives used in the present study, despite being sourced from different suppliers, exhibit comparable thermal performance characteristics.

In series HL2, for which the water content was consistent with that used in studies [43,49], the reported densities ranged from 508 to 627 kg/m^3^, underscoring the role of sample compaction during formation. As with density, thermal conductivity was significantly higher than in HL2 of the present study, reaching values between 0.117 and 0.138 W/m·K. Studies [43,49] presented results for samples with identical shive, binder, and water proportions as in HL2. The higher densities (508–627 kg/m^3^) obtained by the authors indicate that the compaction method notably influenced both density and, consequently, thermal conductivity.

In all series analyzed, the increase in thermal conductivity due to differences in shive granulometry did not exceed 8%, with an average of 4.1%. A clear trend was observed: Finer aggregate fractions led to higher composite density, which in turn resulted in higher thermal conductivity. This trend is consistent with other studies, which show that shive particle size has only a modest impact on the thermal insulation properties of hemp–lime composites [4,6,9,10,11].

All particle size groups used in the present study (F, M, and C) had mean surface areas between 21 and 36 mm^2^, with average lengths exceeding 9 mm (see Table 4). Most studies in the literature report much smaller particle sizes [4,6,9,10,11]. Even the finest fraction used in this study (F) was larger than the coarsest fraction reported in [7], which compared 13 types of hemp shives produced in Europe. This suggests that hemp shives used in construction in countries like France are significantly more finely processed. One contributing factor may be the removal of fine particles and dust during the sieving process, which increased the mean particle size. Nevertheless, this effect was secondary, as the quantity of rejected particles was small.

Elongation and circularity values were similar across all analyzed shive groups, except for the coarsest fraction, which exhibited higher elongation. This can be attributed to longer particles typically having more elongated shapes. However, this is not universally applicable. Some studies have shown that even small particles may have low circularity—around 0.30–0.36 for particles shorter than 7 mm [35]. Similar findings were reported in [16], where approximately 60% of particles had a circularity below 0.27. These observations indicate the wide variability in the shape of hemp shives, which can range from fine and highly elongated to more compact particles with similar length-to-width ratios.

**Figure 14:**
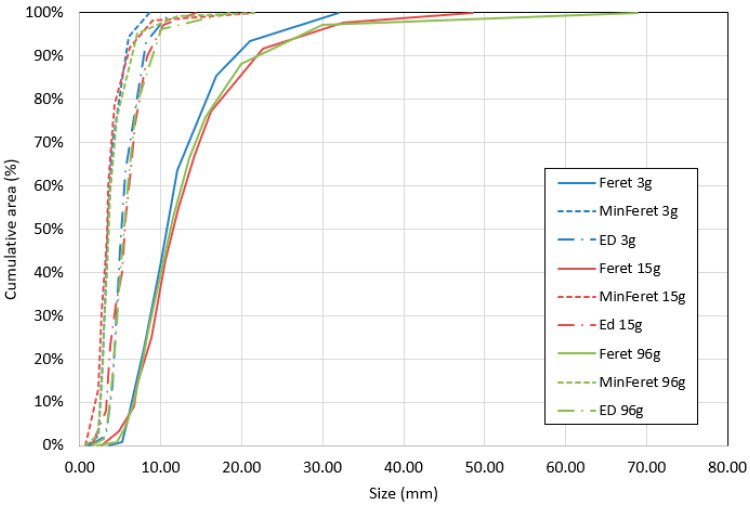
Particle size distribution curves for different sample sizes of medium hemp shives.

To evaluate the accuracy of image analysis results, a comparative assessment was conducted using one randomly selected scan and five randomly selected scans of medium-sized hemp shives (M), and the results were compared with those obtained from the full dataset of 32 scans for this granulometric group (Figure 14). The smallest analyzed sample—3 g—proved insufficient for accurately representing the particle size distribution. This sample contained approximately 500 particles, which does not meet the RILEM technical committee’s recommendation of analyzing a 3–6 g sample containing at least 2000 particles [42]. This requirement was satisfied when five scans of hemp shives were selected.

The total mass of the sample was 15 g, comprising 2416 particles. The analysis conducted on this number of particles yielded results comparable to those obtained from the complete dataset of 32 scans, covering a total of 96 g of material. This indicates that a 15 g sample is sufficient to provide a fast and accurate representation of the particle size distribution for a given hemp shive fraction. The graph (Figure 14) clearly shows differences in the representation of long particles between the 3 g scan and the 15 g and 96 g samples. In a 3 g sample comprising only 500 particles, large particles may be overrepresented due to their disproportionate contribution to the total mass. Given the average size of the hemp shive particles used in this study, granulometric characterization should be based on a minimum 15 g sample to ensure adequate representation of all particle sizes. Larger image-analysis samples, such as 20 g, are not necessary if the average particle length does not exceed 10 mm.

In samples containing only 500 particles, overrepresentation of large particles may occur, as their higher mass results in a disproportionate share of the total 3 g sample weight. Based on the average particle size of the hemp shives analyzed in this study, a minimum sample mass of 15 g is recommended for reliable granulometric analysis. Consequently, the 20 g sample size used in other studies [4,40] appears excessive, particularly when the mean particle length is below 10 mm. To facilitate rapid image-based analysis, the authors recommend using 15 g samples.

## 5. Conclusions and Recommendations

In the conducted study, a clear relationship was observed between the particle size of hemp shives and the thermal conductivity of hemp–lime composites. As the average particle length increased, a systematic decrease in thermal conductivity (λ) was recorded—up to 7.6% between fine and coarse fractions. Simultaneously, the composite density decreased, confirming the influence of granulometry on the material’s microstructure. However, the binder-to-shive ratio had the greatest impact on λ, with an increase in this ratio resulting in over a 20% rise in thermal conductivity, regardless of the shive fraction.

Increasing the water content also led to higher λ values, particularly for fine aggregates (up to 5.7%), which can be attributed to easier compaction of the mixture. All samples in this study were compacted using a constant compaction level of 150% (relative to the loose bulk volume), ensuring consistency and enabling meaningful internal comparison of results.

In contrast, a review of the literature indicates that many published values—even for similar material compositions—can vary significantly due to differences in compaction methods. This suggests that inconsistencies in reported thermal properties across studies are likely influenced more by the sample preparation procedure than by minor variations in mix composition.

Image analysis showed that a 15 g sample (approx. 2400 particles) is sufficient for accurate granulometric characterization. Smaller samples (e.g., 3 g) may lead to statistical bias, particularly the overrepresentation of large particles. Therefore, for hemp shives with an average length below 10 mm, 15 g scans are recommended as the most efficient and reliable method for size distribution analysis.

## Figures and Tables

**Table 1:** Chemical composition of hemp shives by harvest and decortication method (France: mature, dried, no retting; Germany: field-dried before harvest; UK: early harvest with field retting) [13].

Component (% of Dry Mass)	Hemp Shives from France	Hemp Shives from Germany	Hemp Shives from the United Kingdom
Cellulose	47.3	45.6	49.2
Hemicellulose	18.3	17.8	21
Lignin	21.8	23.3	21.9
Extractives	6	5.1	6.2
Others (including ash)	10.3	10.7	5.1

**Table 3:** Methods for determining hemp shive characteristics in scientific articles.

Method	Reference
Dried and dedusted hemp shives were sieved into granulometric groups: 0.5–2 mm, 2–4 mm, 4–8 mm, and 8–16 mm.	[32]
A 10 g sample of hemp shives was photographed using a digital camera. After applying a threshold filter and creating a binary image, particle length and width were measured using ImageJ. The width/length ratio was reported.	[33]
Dried 100 g of hemp shives were sieved, and the percentage of particles smaller than 2.36 mm (87%) and in the range of 2.36–6.3 mm (10%) was determined according to ASTM C136–06.	[34]
According to the RILEM TC 236-BBM protocol, a 3–6 g sample of oven-dried hemp shives (containing at least 2000 particles) is evenly spread on a scanner to prevent particle overlap. An 8-bit grayscale image with 600 dpi resolution is captured and analyzed using ImageJ software. The equivalent diameter and elongation are reported.	[16,35,36,41]
Sieve analysis and 2D image analysis were performed on a 3 g sample using ImageJ software. The equivalent diameter was reported.	[38]
A 112 g sample was sieved for 20 min using sieves with mesh sizes of 8, 4, 2, 1, and 0.5 mm. Subsequently, a 14.6 g sub-sample was manually sorted using a caliper into the following size fractions: <8 mm, 8–16 mm, 16–32 mm, and >32 mm.	[39]
At least 1000 particles from each sample—10 g for coarse aggregate and 5 g for fine aggregate—were measured using an electronic caliper. Both length and width of the hemp shive fragments were recorded.	[9]
The length and width of the particles were measured using 2D image analysis on a 20 g sample.	[40]
A 20 g sample was scanned in batches against a blue background at a resolution of 1200 dpi. All images were processed using ImageJ software. To estimate the aggregate volume, it was assumed that the average particle thickness is proportional to its width. The width/length ratio was reported.	[4]
Samples of 3 g for each type of hemp shive were scanned and analyzed using ImageJ software. The elongation of the particles was reported.	[7]

**Table 4:** Characteristics of hemp shive groups used in the study.

Hemp Shive Group	Bulk Density [kg/m^3^]	Number of Particles	Mean Feret Diameter [mm]	Mean Minimum Feret Diameter [mm]	Mean Equivalent Diameter (ED) [mm]	Mean Area [mm^2^]	Mean Circularity [-]	Mean Elongation [-]
Fine	108.6	19,841	9.26	3.15	4.47	21.33	0.48	3.09
Medium	103.8	17,114	10.02	3.42	4.83	25.52	0.49	3.10
Coarse	97.5	10,536	12.34	4.01	5.79	36.49	0.45	3.33

**Table 5:** The composite formulation (by mass) defines the weight ratio of binder, hemp shives, and water. The letters F, M, and C in the mixture names indicate the use of fine (F), medium (M), and coarse (C) aggregate, respectively.

Series	Mixtures	Hemp Shives	Binder	Water
HL.1	HL1.F HL1.M HL1.C	1	2	2.74
HL.2	HL2.F HL2.M HL2.C	1	2	3.14
HL3	HL3.F HL3.M HL4.C	1	3	3.96

## Data Availability

The data presented in this study are openly available in Zenodo at https://zenodo.org/records/15664512 (accessed on 22 July 2025), reference number 15664512.
